# Spectrum Handoffs Based on Preemptive Repeat Priority Queue in Cognitive Radio Networks

**DOI:** 10.3390/s16071127

**Published:** 2016-07-20

**Authors:** Xiaolong Yang, Xuezhi Tan, Liang Ye, Lin Ma

**Affiliations:** Communication Research Center, Harbin Institute of Technology, Harbin 150080, China; xiaolongyang_hit@163.com (X.Y.); coldwound@163.com (L.Y.); malin@hit.edu.cn (L.M.)

**Keywords:** cognitive radio networks, spectrum handoff, sojourn time, preemptive repeat priority queuing, extended data delivery time, admission control

## Abstract

Cognitive radio can significantly improve the spectrum efficiency, and spectrum handoff is considered as an important functionality to guarantee the quality of service (QoS) of primary users (PUs) and the continuity of data transmission of secondary users (SUs). In this paper, we propose an analytical framework based on a preemptive repeat identical (PRI) M/G/1 queuing network model to characterize spectrum handoff behaviors with general service time distribution of both primary and secondary connections, multiple interruptions and transmission delay resulting from the appearance of primary connections. Then, we derive the close-expression of the extended data delivery and the system sojourn time in both staying and changing scenarios. In addition, based on analysis of spectrum handoff behaviors resulting from multiple interruptions caused by the appearance of the primary connections, we investigate the traffic-adaptive policy, by which the considered SU will optimally adjust its handoff spectrum policy. Moreover, we investigate the admissible region and provide the reference for designing the admission control rule for the arriving secondary connection requests. Finally, simulation results verify that our proposed analytical framework is reasonable and can provide the reference for executing the optimal spectrum handoff strategy and designing the admission control rule for the SU in cognitive radio networks.

## 1. Introduction

Cognitive radio (CR) has been put forward for more than ten years for its promising future, which could significantly improve the spectrum efficiency by allowing the secondary users (SUs) to access the spare licensed spectrum of the primary users (PUs) or the unlicensed spectrum opportunistically [1,2]. However, the SU will impose challenges for preserving the desired quality of service (QoS) of the PU, mainly including four aspects: spectrum sensing, spectrum decision, spectrum sharing, and spectrum mobility [3,4,5]. For the high fluctuation in dynamic CR networks, the SU may be interrupted by the appearance of the PU or the awful channel condition. Therefore, spectrum mobility is an important feature that will guarantee continuous SU data transmission [6,7]. Considered as the main issue in spectrum mobility, spectrum handoff is the process that a SU stays or changes its operating channel upon the appearance of a PU and then re-builds a new communication connection to resume or repeat its data transmission.

Generally, in cognitive radio networks, there are mainly two spectrum handoff types: the reactive approach and the proactive approach depending on the handoff decision time instant [8,9,10]. In the reactive approach, the SU will execute spectrum sensing and resume or repeat its data transmission on a sensed idle channel after handoff triggering factor occurs. The SU will make the handoff decision when interrupted. Although this process can make sure that the SU can find an available channel to continue its data transmission, it will cause a large delay for sensing different channels [11,12]. In the proactive approach, the SU will make the handoff decision before being interrupted. This means that the SU will switch to the prior determined target channel when the handoff triggering factor happens. This approach extremely decreases the delay without spectrum sensing, but it may cause an obsolescence problem that the prior decided target channel is no longer available [13,14]. In other words, these two approaches have their own advantages and disadvantages.

According to different triggering factors, the spectrum handoff modeling techniques are categorized into connection-based and slot-based [15]. In the context of the connection-based spectrum handoffs, the effects of multiple interruptions resulting from PUs are considered in an event-driven manner. By contract, the slot-based modeling technique characterizes multiple interruptions to the SUs in a time-driven manner. That is, the appearance of the PU will trigger the spectrum handoff process in the connection-based scheme, whereas for the slot-based scheme every time slot can be a triggering factor to start to perform a spectrum handoff.

In [15], the preemptive resume priority (PRP) M/G/1 queuing network model was proposed to characterize the connection-based spectrum handoff behaviors with multiple interruptions in multiple-channel CR networks, where the SU can significantly reduce the extended data delivery time by applying the traffic-adaptive target channel sequence selection method. In [16,17,18,19,20,21,22,23], multiple interruptions and the extended data delivery time were also studied in the PRP M/G/1 queuing network model. However, the authors only considered a single-channel CR network. In [24,25,26,27,28], an optimal proactive target channel sequence selection algorithm was proposed to minimize the cumulative spectrum handoff delay in multiple-channel CR networks. A low-complexity greedy algorithm was also investigated based on the trellis diagram. However, it supposed that the newly arriving secondary connection was capable of calculating its optimal target channel sequence while the existing secondary connections did not change their predetermined target channel sequences. Obviously, this assumption neglected the effects to the existing secondary connections, which were caused by the newly arriving traffic loads. In [29], a probabilistic selection algorithm was proposed to predetermine the initial and target channel sequence for a considered secondary connection, which applied the convex optimization theory to minimize the secondary connection extended service and sojourn time. To characterize the multiple-channel CR networks, a queuing analytical framework was built based on the PRP Geom/Geom/1 queuing system model and two policies, which were “stay” and “change”, respectively. In order to reduce the complexity of the algorithm, it proposed the equiprobable target channel selection and the primary traffic-based target channel selection, which were not optimal selection schemes for the considered secondary connection. In general, in [11,12,13,14,15,16,17,18,19,20,21,22,23,24,25,26,27,28,29], all the CR networks were established based on the preemptive resume priority queuing system model. That is, when the secondary connection was interrupted due to the appearance of the primary connection, the interrupted secondary connection would reactively or proactively determine a best available channel to resume its unfinished data transmission. However, in some other scenarios, the interrupted secondary connection may adopt the preemptive repeat scheduling discipline rather than the preemptive resume one. The preemptive repeat priority queue is divided into the preemptive repeat identical (PRI) and the preemptive repeat different (PRD) priority queue. Since the PRD could be viewed as a “simplified” version of the PRI priority queue [30], we focus on the PRI priority queue in this paper.

The PRI priority scheduling discipline was used in the carrier sense multiple access collision detection (CSMA-CD) protocols to describe a fiber optical bus network, which connected a general finite number of stations [31]. Each station with an infinite queuing capacity had different priorities to access the bus network and possibly overwrite information of the downstream stations. By using the randomization solution form and lattice path combinatorics, the transient probability was analyzed for a PRI M/M/1 queuing mode in [32]. The PRI priority queue also was applied to establish an un-slotted optical metropolitan area network (MAN) ring operating with asynchronous variable length optical packets [33,34]. In a ring-based local area network (LAN), a preemptive repeat protocol was proposed to characterize voice-data integration [35], where voice stations had the priority of accessing the ring network. That is, voice stations could overwrite the information of the data stations. Similarly, in some scenarios, the PRI priority scheduling discipline is also worthy of investigating in CR networks. To the best of our knowledge, the preemptive repeat scheduling discipline for characterizing spectrum handoff behaviors in CR networks with multiple interruptions has rarely been studied.

In this paper, unlike [11,12,13,14,15,16,17,18,19,20,21,22,23,24,25,26,27,28,29], we propose an analytical framework based on the preemptive repeat identical (PRI) M/G/1 queuing network model to characterize spectrum handoff behaviors with general service time distribution of both primary and secondary connections, multiple interruptions and transmission time delay resulting from the appearance of primary connections. Under the preemptive repeat policy, when interruptions happen, the secondary connection will start from the beginning. In other words, the data that have been transmitted prior to the interruption are abandoned. The contributions of this paper can be summarized in the following:

(1) We propose an analytical framework based on the PRI M/G/1 queuing network model to analyze spectrum handoff behaviors, and derive the close-expression of extended data delivery and sojourn time in both staying and changing scenarios, respectively.
(2) Based on analysis of spectrum handoff behaviors resulting from multiple interruptions caused by the appearance of the primary connections, we investigate the traffic-adaptive policy, by which the considered SU will optimally adjust its handoff spectrum policy (i.e., the staying policy and the changing policy) to reduce the delay resulting from the appearance of primary connections.
(3) Finally, we investigate the admissible region and provide a reference for designing the admission control rule for the arriving secondary connection requests.

The rest of this paper is organized as follows. Section 2 describes the system model and assumptions. Section 3 analyzes the transmission delay caused by spectrum handoff behaviors in both staying and changing scenarios, and derives the corresponding expressions. Section 4 presents simulation results. Section 5 finally draws a conclusion.

## 2. System Model Description

In this paper, we consider a time-slotted CR network with $N_{c}$ independent primary channels, based on the PRI M/G/1 queuing network model. Each channel has two kinds of queues: high-priority and the low-priority queue. The PUs and SUs enter the high-priority queue and the low-priority queue, respectively, and build corresponding primary and secondary connections. The arrival processes of the primary and secondary connections at channel k are assumed to be Poisson processes with rates $\lambda_{p}^{k}$ and $\lambda_{s}^{k}$, respectively. Furthermore, let random valuables $X_{p}^{k}$ and $X_{s}^{k}$ denote the general service time distribution, respectively, with the corresponding probability density function (PDF) $f_{p}^{k}(x)$ and $f_{s}^{k}(x)$. In the same priority queue, the user accesses to the channel based on the first-come-first-served (FCFS) discipline and will transmit data after the initial handshaking at its default initial channel. Since the PU has the preemptive priority, the SU in low-priority queue can access the channel to transmit data only when the high-priority queue is empty. In the case that the secondary connection is interrupted, the SU will stay on the current operating channel or switch to the target channel to repeat its data transmission.

In addition, each SU is equipped with two kinds of antennas: the directional antenna and the omnidirectional antenna. The directional antenna is used for transmitting or receiving data, whereas the omnidirectional antenna is capable of sensing whether the operating channel is busy or not. To avoid causing harmful interference to the PUs, the listen-before-talk access scheme in the IEEE 802.22 standard [36] is adopted. In this scheme, the SU must perform spectrum sensing at the beginning of each time slot and then transmits its data transmission in the rest of the slot only when the operating channel is detected to be idle. For simplicity, the spectrum sensing is supposed to be perfect without missed detections or false alarms as in [11,12,13,14,15,16,17,18,19,20,21,22,23,24,25,26,27,28,29]. Note that a channel can only be occupied by one user at any time.

### 2.1. Staying Policy

**Figure 1:**
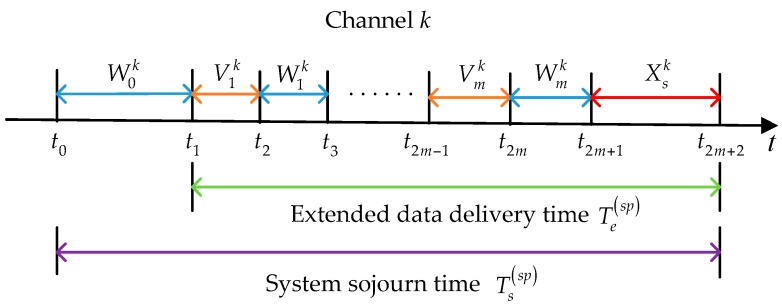
The behaviors of a secondary user (SU) under the staying policy in the preemptive repeat identical (PRI) M/G/1 queuing network model.

Firstly, we analyze the behaviors of a considered SU under the staying policy. As shown in Figure 1, at time instant $t_{0}$, the SU has to enter the low-priority queue of its default initial channel k, which is predetermined by the loading balancing spectrum decision [37] or the optimal probabilistic initial channel selection method [29]. In the staying policy, the SU will always stay in this channel until its data transmission is completed. Let $W_{0}^{k}$ denote the duration from the instant when the SU enters the low-priority queue of channel k to the instant when it transmits data for the first time, and $V_{i}^{k}$ denote the duration that elapses from the instant when the SU starts to transmit its data until it is interrupted by the PU for the *i*th time. In the PRI M/G/1 queuing network model, the effective service time $V_{i}^{k}$ is lost in the repeat scheduling discipline. Note that the SU completes its data transmission after experiencing m interruptions. Denote $W_{i}^{k}$ as the service time for the newly arriving PU at the *i*th interruption. Thus, we have:

(1) $$T_{e}^{\left(sp\right)}=X_{s}^{k}+\sum_{i=1}^{m}\left(V_{i}^{k}+W_{i}^{k}\right)=X_{s}^{k}+V^{k}+W^{k}$$

where $T_{e}^{\left(sp\right)}$ represents the extended data delivery time in the staying policy, and:

(2) $$T_{s}^{\left(sp\right)}=T_{e}^{\left(sp\right)}+W_{0}^{k}$$

where $T_{s}^{\left(sp\right)}$ denotes the system sojourn time in the staying policy from the instant when the SU enters the low-priority queue to the instant when its data transmission is completed.

### 2.2. Changing Policy

Then, we analyze the behaviors of the considered SU under the changing policy. In this scenario, we assume that the target channel sequence with initial channel θ is denoted as $C^{\left(\theta \right)}=\left(c_{1,\theta },c_{2,\theta },\cdots ,c_{i,\theta },\cdots c_{M,\theta }\right)$, $1\leq c_{i,\theta }\leq N_{c}$, 1≤i≤M, where $c_{i,\theta }$ is the target channel at the *i*th interruption and M is defined as the maximum allowable interruption times. In the proactive spectrum handoff process, the target channel sequence is determined before data transmission. Note that in the changing policy $c_{i,\theta }\neq c_{i-1,\theta }$ as in [15,29]. In other words, the SU will switch to the target channel as long as the interruption happens. If the target channel is idle, the SU can execute data transmission immediately. Otherwise, according to the proactive spectrum handoff discipline, the SU has to wait until the channel is idle.

**Figure 2:**
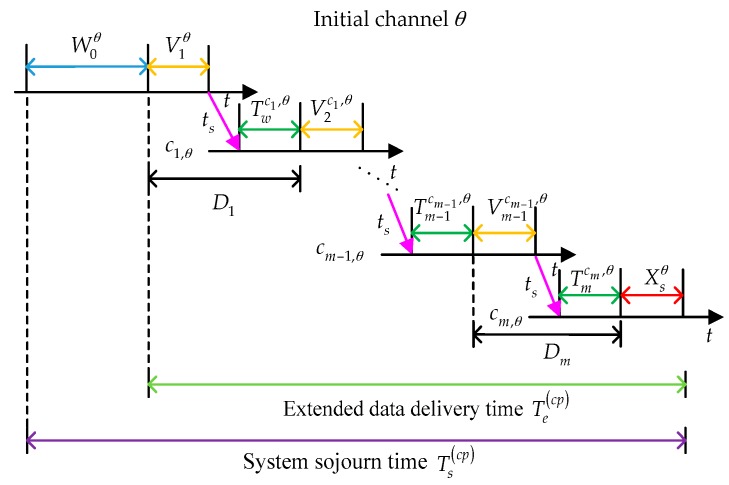
The behaviors of a secondary user (SU) under the changing policy in the preemptive repeat identical (PRI) M/G/1 queuing network model.

The whole proactive spectrum handoff process is illustrated in Figure 2. The considered SU enters the pre-assigned initial channel θ, and has to wait until the initial channel is idle. The waiting time on initial channel θ can be represented as $W_{0}^{\theta }$. From the instant when the considered SU transmits its data to the instant when the SU completes its data transmission, it will encounter multiple interruptions caused by the appearance of the primary connections. As shown in Figure 2, let $T_{w}^{c_{i,\theta }}$ denote the waiting time of the SU before starting data transmission at target channel $c_{i,\theta }$ and $t_{s}$ denote the switching time. Suppose that the SU finishes its data transmission at target channel $c_{m,\theta }\left(1\leq m\leq M\right)$. Then, we have:

(3) $$D_{i}=T_{w}^{c_{i,\theta }}+V_{i}^{c_{i,\theta }}+t_{s},1\leq i\leq m$$

where $D_{i}$ represents the delay caused by the *i*th interruption.

Based on the analysis above, the extended data delivery time under the changing policy can be given by:

(4) $$T_{e}^{\left(cp\right)}=X_{s}^{\theta }+\sum_{i=1}^{m}D_{i}$$

and the system sojourn time can be expressed as:

(5) $$T_{s}^{\left(cp\right)}=T_{e}^{\left(cp\right)}+W_{0}^{\theta }$$

## 3. Analysis and Derivation for Parameters

In this section, we derive the average extended data delivery time $\left(E\left[T_{e}^{\left(sp\right)}\right],E\left[T_{e}^{\left(cp\right)}\right]\right)$ and the system sojourn time $\left(E\left[T_{s}^{\left(sp\right)}\right],E\left[T_{s}^{\left(cp\right)}\right]\right)$ of the SU based on both staying and changing policies, in the PRI M/G/1 queuing network.

### 3.1. Derivation under Staying Policy

Suppose that L is a random variable, which denotes the number of times that the SU has been interrupted until its data transmission is completed. Let $p_{s}^{k}$ represent the probability that the SU enters a channel for service and completes its data transmission without being interrupted. Obviously, L can be seen as a geometrical distribution with the probability mass function:

(6) $$\mathrm{Pr}\left(L=l\right)=p_{s}^{k}{\left(1-p_{s}^{k}\right)}^{l}\;l\geq 0$$

Thus, the average number of times E[L] can be derived as the following:

(7) $$\begin{array}{ll} E\left[L\right] & =\sum_{l=0}^{+\infty }l\mathrm{Pr}\left(L=l\right)\\ & =\sum_{l=1}^{+\infty }lp_{s}^{k}{\left(1-p_{s}^{k}\right)}^{l}\\ & =\frac{1-p_{s}^{k}}{p_{s}^{k}} \end{array}$$

For further derivation, we give two propositions first.

Proposition 1: Given the arrival rate $\lambda_{p}^{k}$ of PUs and the PDF $f_{s}^{k}\left(x\right)$ of the SU service time distribution on channel k, we have $p_{s}^{k}=\int_{0}^{\infty }e^{-\lambda_{p}^{k}x}f_{s}^{k}\left(x\right)dx$.

Proof: According to the assumptions above, the arrivals of PUs occur in a Poisson process. Thus, within time interval [t,t+x], the probability that n PUs arrive on channel k can be given by:

(8) $$P\left[N\left(t+x\right)-N\left(t\right)=n\right]=\frac{e^{-\lambda_{p}^{k}x}{\left(\lambda_{p}^{k}x\right)}^{n}}{n!},\;n=0,1,2,\cdots$$

Obviously, the probability that no PU appears on channel k is $P\left[N\left(t+x\right)-N\left(t\right)=0\right]=e^{-\lambda_{p}^{k}x}$. In addition, $f_{s}^{k}\left(x\right)$ represents the PDF of the service time distribution of the SU on channel k. Therefore, the average probability that the SU can enter for service and complete the service without being interrupted can be expressed as:

(9) $$\begin{array}{ll} p_{s}^{k} & =\int_{0}^{\infty }P\left[N\left(t+x\right)-N\left(t\right)=0\right]f_{s}^{k}\left(x\right)dx\\ & =\int_{0}^{\infty }e^{-\lambda_{p}^{k}x}f_{s}^{k}\left(x\right)dx \end{array}$$

As a result, the conclusion in proposition 1 is obtained.

Proposition 2: Given the general service time distribution $X_{s}^{k}$ and the corresponding PDF $f_{s}^{k}\left(x\right)$ on channel k, we have $E\left[V_{i}^{k}\right]=\frac{E\left[{\left(X_{s}^{k}\right)}^{2}\right]}{2E\left[X_{s}^{k}\right]}$ for ∀i∈{1,2,⋯,M}.

Proof: We prove this conclusion by the renewal theory. Suppose that Y denotes the length of the inter-arrival gap that the PU arrives by random incidence, whose PDF is $f_{Y}^{k}\left(y\right)$. Then, we have:

(10) $$Y=V_{i}^{k}+Z$$

where $V_{i}^{k}$ denotes the effective service time for the SU before interrupted and Z represents the residual time of the inter-arrival gap, whose PDF is $f_{Z}^{k}\left(z\right)$.

Firstly, we derive the expression of E[Y]. The probability that a PU arrival occurs in a gap [y,y+dy] is given by $f_{Y}^{k}\left(y\right)dy$, which can be supposed to be directly proportional to the length of the gap y and relative occurrence $f_{s}^{k}\left(y\right)dy$ of such gap. Thus, we have:

(11) $$f_{Y}^{k}\left(y\right)dy=\xi yf_{s}^{k}\left(y\right)dy$$

where ξ is the proportionality coefficient. Thus,

(12) $$\begin{array}{ll} E\left[Y\right] & =\int_{-\infty }^{+\infty }yf_{Y}^{k}\left(y\right)dy\\ & =\int_{-\infty }^{+\infty }\xi y^{2}f_{s}^{k}\left(y\right)dy\\ & =\xi E\left[{\left(X_{s}^{k}\right)}^{2}\right] \end{array}$$

In addition,

(13) $$\begin{array}{ll} \int_{-\infty }^{+\infty }f_{Y}^{k}\left(y\right)dy & =\int_{-\infty }^{+\infty }\xi yf_{s}^{k}\left(y\right)dy\\ & =\xi E\left[X_{s}^{k}\right]\\ & =1 \end{array}$$

Comparing Equations (12) and (13), we have:

(14) $$E\left[Y\right]=\frac{E\left[{\left(X_{s}^{k}\right)}^{2}\right]}{E\left[X_{s}^{k}\right]}$$

Then, we need to derive the express of E[Z]. For the given gap length y, the PU can arrive at anywhere within the gap. Thus, the conditional PDF of Z is given by:

(15) $$f_{Z|Y}^{k}\left(z|y\right)=\frac{1}{y},0\leq z\leq y$$

Therefore, we have the marginal PDF of Z:

(16) $$\begin{array}{ll} f_{Z}^{k}\left(z\right) & =\int_{-\infty }^{+\infty }f_{ZY}^{k}\left(z,y\right)dy\\ & =\int_{-\infty }^{+\infty }f_{Y}^{k}\left(y\right)f_{Z|Y}^{k}\left(z|y\right)dy\\ & =\int_{z}^{+\infty }\xi yf_{s}^{k}\left(y\right)\frac{1}{y}dy\\ & =\int_{z}^{+\infty }\frac{f_{s}^{k}\left(y\right)}{E\left[X_{s}^{k}\right]}dy,0\leq z \end{array}$$

Then, we have:

(17) $$\begin{array}{ll} E\left[Z\right] & =\int_{0}^{+\infty }zf_{Z}^{k}\left(z\right)dz\\ & =\frac{1}{E\left[X_{s}^{k}\right]}\int_{z=0}^{+\infty }z\int_{y=z}^{+\infty }f_{s}^{k}\left(y\right)dydz\\ & =\frac{1}{E\left[X_{s}^{k}\right]}\int_{y=0}^{+\infty }\int_{z=0}^{y}zf_{s}^{k}\left(y\right)dzdy\\ & =\frac{1}{E\left[X_{s}^{k}\right]}\int_{y=0}^{+\infty }\frac{1}{2}y^{2}f_{s}^{k}\left(y\right)dy\\ & =\frac{E\left[{\left(X_{s}^{k}\right)}^{2}\right]}{2E\left[X_{s}^{k}\right]} \end{array}$$

Finally, substituting Equation (17) into Equation (10), we can obtain:

(18) $$E\left[V_{i}^{k}\right]=E\left[Y\right]-E\left[Z\right]=\frac{E\left[{\left(X_{s}^{k}\right)}^{2}\right]}{2E\left[X_{s}^{k}\right]}$$

Based on Equations (1), (7) and (18), the lost effective service time under the staying policy can be expressed as:

(19) $$E\left[V^{k}\right]=E\left[L\right]E\left[V_{i}^{k}\right]=\frac{1-p_{s}^{k}}{p_{s}^{k}}\frac{E\left[{\left(X_{s}^{k}\right)}^{2}\right]}{2E\left[X_{s}^{k}\right]}$$

In addition, $E\left[W^{k}\right]$ represents the mean time to serve the PU who arrives within the average extended data delivery time duration. Thus,

(20) $$E\left[W^{k}\right]=\lambda_{p}^{k}E\left[X_{p}^{k}\right]E\left[T_{e}^{\left(sp\right)}\right]$$

where the average extended data delivery time is given by:

(21) $$E\left[T_{e}^{\left(sp\right)}\right]=E\left[X_{s}^{k}\right]+E\left[V^{k}\right]+E\left[W^{k}\right]$$

Substituting Equations (19) and (20) into Equation (21), we have:

(22) $$E\left[T_{e}^{\left(sp\right)}\right]=\frac{1}{1-\lambda_{p}^{k}E\left[X_{p}^{k}\right]}\left(E\left[X_{s}^{k}\right]+\frac{1-p_{s}^{k}}{p_{s}^{k}}\frac{E\left[{\left(X_{s}^{k}\right)}^{2}\right]}{2E\left[X_{s}^{k}\right]}\right)$$

Similarly,

(23) $$E\left[W_{0}^{k}\right]=E\left[{\tilde{W}}_{0}^{k}\right]+\lambda_{p}^{k}E\left[X_{p}^{k}\right]E\left[W_{0}^{k}\right]$$

where $E\left[{\tilde{W}}_{0}^{k}\right]$ denotes the mean time to serve the PUs and SUs that are ahead of the considered SU and can be given by [38]:

(24) $$E\left[{\tilde{W}}_{0}^{k}\right]=\frac{\lambda_{s}^{k}E\left[{\left(X_{s}^{k}\right)}^{2}\right]+\lambda_{p}^{k}E\left[{\left(X_{p}^{k}\right)}^{2}\right]}{2\left(1-\lambda_{s}^{k}E\left[X_{s}^{k}\right]-\lambda_{p}^{k}E\left[X_{p}^{k}\right]\right)}$$

and $\lambda_{p}^{k}E\left[W_{0}^{k}\right]$ denotes the number of PUs those arrive in the average time $E\left[W_{0}^{k}\right]$. Thus, $\lambda_{p}^{k}E\left[X_{p}^{k}\right]E\left[W_{0}^{k}\right]$ is the mean time to serve the newly arriving PUs. Based on Equations (2) and (22)–(24), the average system sojourn time in the staying policy can be finally given by:

(25) $$E\left[T_{s}^{\left(sp\right)}\right]=\frac{E\left[X_{s}^{k}\right]}{1-\rho_{p}^{k}}+\frac{1-p_{s}^{k}}{p_{s}^{k}}\frac{E\left[{\left(X_{s}^{k}\right)}^{2}\right]}{2\left(1-\rho_{p}^{k}\right)E\left[X_{s}^{k}\right]}+\frac{\lambda_{s}^{k}E\left[{\left(X_{s}^{k}\right)}^{2}\right]+\lambda_{p}^{k}E\left[{\left(X_{p}^{k}\right)}^{2}\right]}{2\left(1-\rho_{p}^{k}\right)\left(1-\rho_{s}^{k}-\rho_{p}^{k}\right)}$$

where $\rho_{p}^{k}=\lambda_{p}^{k}E\left[X_{p}^{k}\right]$ and $\rho_{s}^{k}=\lambda_{s}^{k}E\left[X_{s}^{k}\right]$ can be interpreted as the primary and secondary traffic intensity resulting from primary and secondary connections of channel k, respectively. For a stable queuing system, we have $\rho_{p}^{k}+\rho_{s}^{k}<1$. In other words, the utilization factor in each channel must be less than 1.

### 3.2. Derivation under Changing Policy

In this part, the considered SU will adopt the changing policy as shown in Figure 2 when interruption happens. Firstly, we consider two important system parameters: $\psi_{i}^{c_{i,\theta }}$ and $\Psi_{i}^{c_{i,\theta }}$. $\psi_{i}^{c_{i,\theta }}$ is defined as the arrival rate, at channel $c_{i,\theta }$, of the secondary connections those has experienced *i* interruptions and $\Psi_{i}^{c_{i,\theta }}$ is defined as the transmission duration of a secondary connection between the *i*th and the (*i* + 1)th interruption. Thus, referring to [15], we have:

(26) $$\begin{array}{ll} E\left[T_{w}^{c_{i,\theta }}\right] & =\frac{\lambda_{p}^{c_{i,\theta }}E\left[{\left(X_{p}^{c_{i,\theta }}\right)}^{2}\right]+\lambda_{s}^{c_{i,\theta }}E\left[{\left(X_{s}^{c_{i,\theta }}\right)}^{2}\right]+\sum_{i=1}^{M}\psi_{i}^{c_{i,\theta }}E\left[{\left(\Psi_{i}^{c_{i,\theta }}\right)}^{2}\right]}{2\left(1-\rho_{p}^{c_{i,\theta }}-\rho_{s}^{c_{i,\theta }}-\sum_{i=1}^{M}\psi_{i}^{c_{i,\theta }}E\left[\Psi_{i}^{c_{i,\theta }}\right]\right)}\\ & +\frac{{\left(\lambda_{p}^{c_{i,\theta }}\right)}^{2}E\left[{\left(X_{p}^{c_{i,\theta }}\right)}^{2}\right]E\left[X_{p}^{c_{i,\theta }}\right]}{2\left(1-\rho_{p}^{c_{i,\theta }}-\rho_{s}^{c_{i,\theta }}-\sum_{i=1}^{M}\psi_{i}^{c_{i,\theta }}E\left[\Psi_{i}^{c_{i,\theta }}\right]\right)\left(1-\rho_{p}^{c_{i,\theta }}\right)} \end{array}$$

Note that the average waiting time $E\left[T_{w}^{c_{i,\theta }}\right]$ is influenced by the arrival rate of the interrupted secondary connections and their corresponding effective service time duration, except for the arrival rate and service time distribution of the primary and secondary connections at target channel $c_{i,\theta }$. In addition, for a stable queuing system, we have:

(27) $$\rho_{p}^{c_{i,\theta }}+\rho_{s}^{c_{i,\theta }}+\sum_{i=1}^{M}\psi_{i}^{c_{i,\theta }}E\left[\Psi_{i}^{c_{i,\theta }}\right]<1$$

Based on proposition 2, we have $V_{i}^{c_{i,\theta }}=E\left[{\left(X_{s}^{c_{i,\theta }}\right)}^{2}\right]/2E\left[X_{s}^{c_{i,\theta }}\right]$. Thus, the average delay caused by the *i*th interruption can be given by:

(28) $$E\left[D_{i}\right]=E\left[T_{w}^{c_{i,\theta }}\right]+\frac{E\left[{\left(X_{s}^{c_{i,\theta }}\right)}^{2}\right]}{2E\left[X_{s}^{c_{i,\theta }}\right]}+t_{s}$$

Recall the defined random variable L, which denotes the number of times that the SU is interrupted until its data transmission is completed. The average total delay can be expressed as:

(29) $$E\left[D\right]=\sum_{m=1}^{M}\left[\mathrm{Pr}\left(L=m\right)\sum_{i=1}^{m}E\left[D_{i}\right]\right]$$

where Pr(L=m) denotes the probability that the considered SU has been interrupted m times until its data transmission is completed. Thus, we have:

(30) $$\mathrm{Pr}\left(L=m\right)=\int_{0}^{\infty }e^{-\lambda_{p}^{c_{m,\theta }}x}f_{s}^{c_{m,\theta }}\left(x\right)dx\prod_{i=0}^{m-1}\left(1-\int_{0}^{\infty }e^{-\lambda_{p}^{c_{i,\theta }}x}f_{s}^{c_{i,\theta }}\left(x\right)dx\right)$$

Finally, according to Equation (4), we have:

(31) $$E\left[T_{e}^{\left(cp\right)}\right]=E\left[X_{s}^{\theta }\right]+E\left[D\right]$$

Thus, the average extended data delivery time based on the changing policy is given by:

(32) $$\begin{array}{ll} E\left[T_{e}^{\left(cp\right)}\right]= & E\left[X_{s}^{\theta }\right]+\sum_{m=1}^{M}[\int_{0}^{\infty }e^{-\lambda_{p}^{c_{m,\theta }}x}f_{s}^{c_{m,\theta }}\left(x\right)dx\prod_{i=0}^{m-1}\left(1-\int_{0}^{\infty }e^{-\lambda_{p}^{c_{i,\theta }}x}f_{s}^{c_{i,\theta }}\left(x\right)dx\right)\\ & \sum_{i=1}^{m}(\frac{E\left[{\left(X_{s}^{c_{i,\theta }}\right)}^{2}\right]}{2E\left[X_{s}^{c_{i,\theta }}\right]}+t_{s}+\frac{\lambda_{p}^{c_{i,\theta }}E\left[{\left(X_{p}^{c_{i,\theta }}\right)}^{2}\right]+\lambda_{s}^{c_{i,\theta }}E\left[{\left(X_{s}^{c_{i,\theta }}\right)}^{2}\right]+\sum_{i=1}^{M}\psi_{i}^{c_{i,\theta }}E\left[{\left(\Psi_{i}^{c_{i,\theta }}\right)}^{2}\right]}{2\left(1-\rho_{p}^{c_{i,\theta }}-\rho_{s}^{c_{i,\theta }}-\sum_{i=1}^{M}\psi_{i}^{c_{i,\theta }}E\left[\Psi_{i}^{c_{i,\theta }}\right]\right)}\\ & +\frac{{\left(\lambda_{p}^{c_{i,\theta }}\right)}^{2}E\left[{\left(X_{p}^{c_{i,\theta }}\right)}^{2}\right]E\left[X_{p}^{c_{i,\theta }}\right]}{2\left(1-\rho_{p}^{c_{i,\theta }}-\rho_{s}^{c_{i,\theta }}-\sum_{i=1}^{M}\psi_{i}^{c_{i,\theta }}E\left[\Psi_{i}^{c_{i,\theta }}\right]\right)\left(1-\rho_{p}^{c_{i,\theta }}\right)})] \end{array}$$

Note that selecting different target channel sequences will result in different average extended data delivery times, and an optimal target channel sequence selection can be realized by the dynamic programming algorithm in [35]. Substituting Equation (24) into Equation (5), the average system sojourn time is:

(33) $$E\left[T_{s}^{\left(cp\right)}\right]=E\left[T_{e}^{\left(cp\right)}\right]+\frac{\lambda_{s}^{\theta }E\left[{\left(X_{s}^{\theta }\right)}^{2}\right]+\lambda_{p}^{\theta }E\left[{\left(X_{p}^{\theta }\right)}^{2}\right]}{2\left(1-\rho_{p}^{\theta }\right)\left(1-\rho_{s}^{\theta }-\rho_{p}^{\theta }\right)}$$

### 3.3. Traffic-Adaptive Policy

Generally, the considered SU prefers adopting the spectrum handoff policy that can reduce the average extended data delivery time. That is:

(34) $$E\left[T_{e}^{*}\right]=\min\left(E\left[T_{e}^{\left(sp\right)}\right],E\left[T_{e}^{\left(cp\right)}\right]\right)$$

In other words, if $E\left[T_{e}^{\left(sp\right)}\right]\geq E\left[T_{e}^{\left(cp\right)}\right]$, the SU will adopt the changing policy. If $E\left[T_{e}^{\left(sp\right)}\right]\leq E\left[T_{e}^{\left(cp\right)}\right]$, the SU will adopt the staying policy. This process is called the traffic-adaptive policy. Similarly, for the average system sojourn time, we have:

(35) $$E\left[T_{s}^{*}\right]=\min\left(E\left[T_{s}^{\left(sp\right)}\right],E\left[T_{s}^{\left(cp\right)}\right]\right)$$

For both spectrum handoff polices, the initial channel is pre-assigned. Thus, the minimum average extended data delivery time will result in the minimum average system sojourn time.

## 4. Numerical Results and Discussion

In this paper, the MATLAB simulation platform is used. In order to validate the proposed analytical network model, we consider a three-channel (i.e., $N_{c}=3$) continuous-time cognitive radio system, where a waiting connection (primary or secondary connection) is put into service as soon as the server becomes available. In other words, the average extended data delivery and system sojourn time are non-integer time slots in the continuous-time system. Furthermore, assume that these three channels are identical and the primary and secondary connections have the same traffic parameters, i.e., $\lambda_{p}^{1}=\lambda_{p}^{2}=\lambda_{p}^{3}=\lambda_{p}$, $\lambda_{s}^{1}=\lambda_{s}^{2}=\lambda_{s}^{3}=\lambda_{s}$, and $E[X_{p}^{1}]=E[X_{p}^{2}]=E[X_{p}^{3}]=E[X_{p}]$. Because our analytical framework is established on the PRI M/G/1 queuing network model, the arrival rates of the primary and secondary connections are Poisson arrival rates for the high-priority and low-priority connections, respectively. The primary connection service time follows the general distribution. In addition, suppose that the secondary connection service time follows the exponential distribution. According to this assumption, we have $f_{s}^{1}\left(x\right)=f_{s}^{2}\left(x\right)=f_{s}^{3}\left(x\right)=f_{s}\left(x\right)=\alpha e^{-\alpha x}$. Hence, the average service time for the secondary connection is $E[X_{s}^{1}]=E[X_{s}^{2}]=E[X_{s}^{3}]=E[X_{s}]=1/\alpha$. We also have $p_{s}^{1}=p_{s}^{2}=p_{s}^{3}=p_{s}$, which is given by:

(36) $$p_{s}=\int_{0}^{\infty }e^{-\lambda_{p}x}f_{s}\left(x\right)dx=\frac{\alpha }{\lambda_{p}+\alpha }$$

### 4.1. Effects of Channel Busy Probability $\rho_{p}$ and Average Service Time $E[X_{s}]$ on $p_{s}$

**Figure 3:**
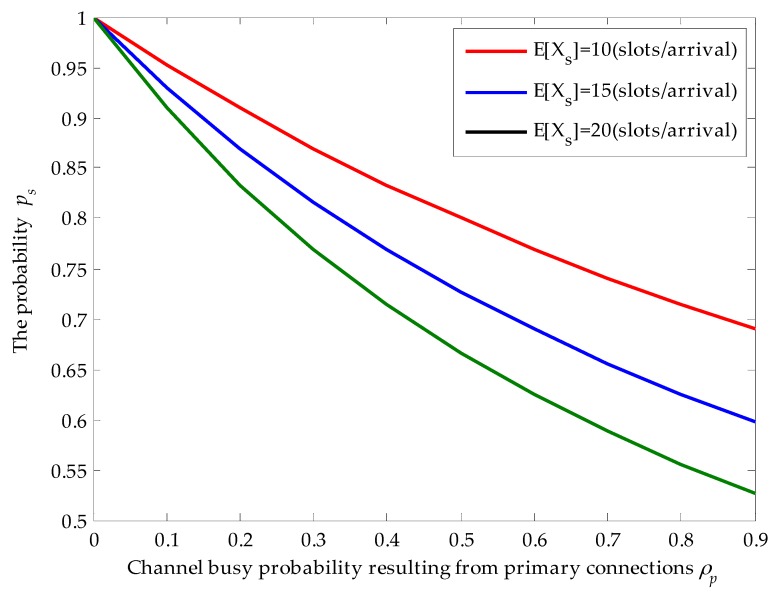
Effects of the channel busy probability $\rho_{p}$ resulting from primary connections and the average service time $E[X_{s}]$ of secondary connections on the probability $p_{s}$, where $E[X_{p}]=20$ (slots/arrival), $\lambda_{s}=0.01$ (arrivals/slot), $\lambda_{p}=0.02$ (arrivals/slot) and $\lambda_{s}E[X_{s}]+\rho_{p}<1$.

Figure 3 shows the effects of channel busy probability $\rho_{p}$ and the average service time of secondary connection on the probability $p_{s}$ when the average service time of primary connections is given. Firstly, we find that the obtained results in Figure 3 match the derivation results. With the increase of channel busy probability $\rho_{p}$, the probability $p_{s}$ that the secondary connection enters the target channel for service and completes its data transmission without being interrupted decreases. For example, given that $E[X_{s}]=20$ (slots/arrival), when $\rho_{p}=0.1$, $p_{s}=0.91$. However, when $\rho_{p}=0.8$, the probability $p_{s}$ dramatically decreases to 0.56. That is because higher channel busy probability will result in larger arrival rates of newly arriving primary connections. Hence, the probability that the secondary connection is interrupted by the newly arriving primary connections becomes larger. In addition, for given channel busy probability, with the increase of average service time of secondary connections, $p_{s}$ also decreases. That is because longer average service time raises the risk that the secondary connection is interrupted by the newly arriving primary connections. For instance, given that $\rho_{p}=0.4$, $p_{s}$ decreases from 0.83 to 0.71 when the average service time of secondary connections changes from 10 (slots/arrival) to 20 (slots/arrival). Thus, the behaviors of the SU depend on the average service time $E[X_{s}]$ of the SU and the channel busy probability $\rho_{p}$ resulting from the primary connections.

### 4.2. Effects of Various Service Time Distributions for Primary Connections

Since the initial channel is pre-assigned by methods in [29,37], the waiting time duration from the instant when the SU enters the low-priority queue to the instant when the SU transmits its data for the first time is determined. Thus, we focus on discussing the effects of various service time distributions for primary connections on the average extended data delivery time. Referring to [39], we consider two kinds of service time distributions for primary connections: the exponential distribution and the upper-truncated Pareto distribution, matching the actual voice and data traffic measurements quite well, respectively. According to [40], the formula for the *g*th moment of the upper-truncated Pareto distribution is given by:

(37) $$E\left[X_{pareto}^{g}\right]=\frac{Q^{\beta }\left(\beta Q^{\left(g-\beta \right)}-gH^{\left(g-\beta \right)}\right)}{\left(\beta -g\right)},\beta \neq g,g=1,2,\cdots$$

where β is the shape parameter; and Q and H denotes the scale parameter and the truncated upper bound, respectively. We set the shape parameter β=1.1, the scale parameter Q=81.5, and the truncated upper bound H=66,666 bytes. Thus, the average connection length of the primary connection is calculated to be 20 (slots/arrival), i.e., $E[X_{p}]=E{\left[X_{Pareto}^{g}\right]}_{g=1}=20$ (slots/arrival) when the slot duration is considered to be 10 millisecond as adopted in the IEEE 802.22 standard and the data rate is set to be 19.2 kbps. In addition, the formula for the *g*th moment of the exponential distribution is expressed as:

(38) $$E\left[X_{expential}^{g}\right]=\frac{g!}{{\left(\mu_{p}\right)}^{g}},g=1,2,\cdots$$

For fair comparison, the average connection length of the primary connection for the exponential distribution is also assumed to be 20 (slots/arrival), i.e., $E{\left[X_{exponential}^{g}\right]}_{g=1}=E{\left[X_{Pareto}^{g}\right]}_{g=1}=20$ (slots/arrival). Furthermore, we suppose that $\lambda_{s}=0.01$ (arrivals/slot), and $E[X_{s}]=10$ (slots /arrival). Note that for a stable queuing system, we have $\lambda_{s}E[X_{s}]+\rho_{p}<1$.

**Figure 4:**
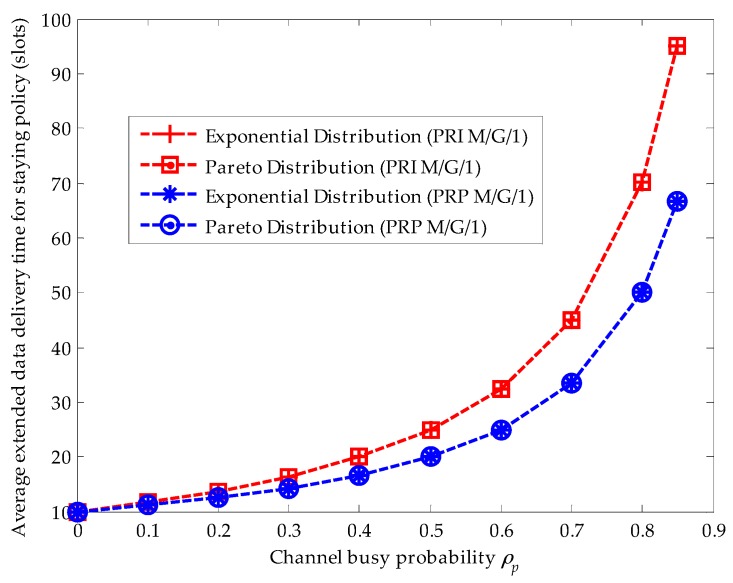
Effects of the upper-truncated Pareto distribution and the exponential distribution for primary connections when the staying policy is adopted in the preemptive repeat identical (PRI) M/G/1 and the preemptive resume priority (PRP) M/G/1 queuing network models.

Figure 4 compares the effects of the upper-truncated Pareto distribution and the exponential distribution for primary connections in the PRI M/G/1 and the PRP M/G/1 analytical frameworks when the staying policy is adopted. Firstly, we can see that the extended data delivery times in both analytical frameworks are the same. Because the extended data delivery time in the staying policy in Equation (22) is determined by the average service time of primary connections and the average service times are the same for the upper-truncated Pareto distribution and the exponential distribution, i.e., $E[X_{p}]=E{\left[X_{exponential}^{g}\right]}_{g=1}=E{\left[X_{Pareto}^{g}\right]}_{g=1}$. Secondly, in the PRI M/G/1 analytical framework, the extended data delivery time is longer than that in the PRP M/G/1 analytical framework for the reason that the SU has to repeat its data transmission when an interruption happens in the PRI M/G/1 queuing network model. In other words, in the PRI M/G/1 queuing network model, the effective service time $V_{i}$ is lost in the repeat scheduling discipline. Moreover, with the increase of the channel busy probability, the difference value of the average service time in these two analytical frameworks becomes larger for the reason that a larger channel busy probability will result in more interruptions. For example, when $\rho_{p}=0.2$, the difference value equals to 1.25 slots. When $\rho_{p}=0.6$, the difference value equals to 7.5 slots.

**Figure 5:**
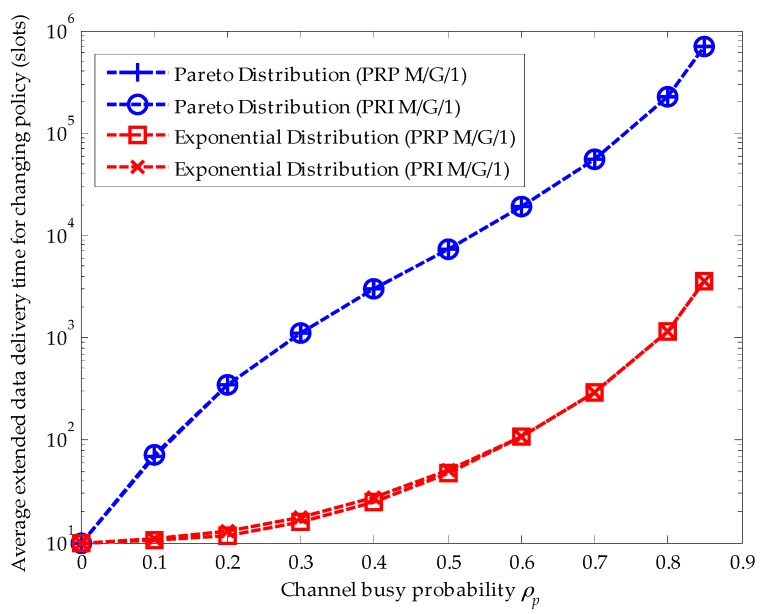
Effects of the upper-truncated Pareto distribution and the exponential distribution for primary connections when the changing policy is adopted in the preemptive repeat identical (PRI) M/G/1 and the preemptive resume priority (PRP) M/G/1 queuing network models, where $\lambda_{s}=0.01$ (arrivals/slot), $E[X_{s}]=10$ (slots/arrival), $t_{s}=1$ and (slot).

Figure 5 compares the effects of the upper-truncated Pareto distribution and the exponential distribution for primary connections in the PRI M/G/1 and the PRP M/G/1 analytical frameworks when the changing policy is adopted. In both analytical frameworks, the upper-truncated Pareto distribution for primary connections results in a larger extended data delivery time than the exponential distribution for primary connections. This is because the second moment $E{\left[X_{Pareto}^{g}\right]}_{g=2}$ of the upper-truncated Pareto distribution is larger than that of the exponential distribution $E{\left[X_{exponential}^{g}\right]}_{g=2}$. Furthermore, in the changing policy, the delay in a secondary connection caused by the repeat scheduling discipline can be omitted for the reason that the second moment of the upper-truncated Pareto distribution and exponential distribution results in a huge delay.

In addition, we also need to discuss two service time distributions for secondary connections: the upper-truncated Pareto distribution and the exponential distribution. However, from the derivation results, we can see that the extended data delivery time is determined by the averge service time, not related to the *g*th (*g* ≥ 2) moment of the service time distribution. Therefore, when the service time distribution of the secondary connection follows the upper-truncated Pareto distribution with $E[X_{s}]=E{\left[X_{exponential}^{g}\right]}_{g=1}=E{\left[X_{Pareto}^{g}\right]}_{g=1}$, we will obtain the same results as Figure 4 and Figure 5. Thus, for simpicity, we omit discussion about the upper-truncated Pareto distribution for secondary connections.

### 4.3. Traffic-Adaptive Policy for the Secondary Connection

**Figure 6:**
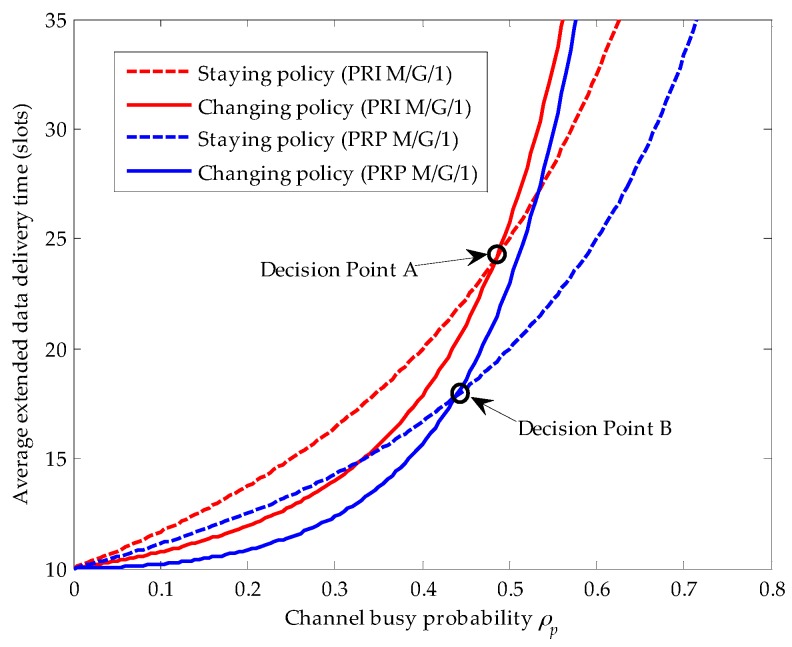
Compares the traffic-adaptive policy of the considered secondary user (SU) in the preemptive repeat identical (PRI) M/G/1 and the preemptive resume priority (PRP) M/G/1 queuing network models, where the primary connection follows the exponential distribution, $\lambda_{s}=0.01$ (arrivals/slot), $E[X_{s}]=10$ (slots/arrival), $0\leq \rho_{p}<0.8$, $t_{s}=1$ (slot) and $\lambda_{s}E[X_{s}]+\rho_{p}<1$.

Comparing Figure 4 with Figure 5, we clearly know that when the service time distribution of the primary connection follows the upper-truncated Pareto distribution, the average extended data delivery time of the secondary connection in the changing policy becomes so large that there is no cross point between the average extended data delivery time curve for the staying policy and that for the changing policy. That is, when the service time distribution of the primary connection follows the upper-truncated Pareto distribution, there is no traffic-adaptive policy for the secondary connection. However, as the service time distribution of the primary connection follows the exponential distribution, the cross point between the staying policy and the changing policy exits, as shown in Figure 6.

In this figure, we define the cross point as the decision point, and let A and B denote the decision point in the PRI M/G/1 and the PRP M/G/1 queuing networks, respectively. In other words, if the channel busy probability resulting from the arrival of the primary connection lies on the left side of decision point A (i.e., $\rho_{p}<0.485$), the secondary connection will execute the changing policy for shorter average extended data delivery time in the PRI M/G/1 queuing network. On the contrary, if $\rho_{p}>0.485$, the secondary connection will adaptively choose the staying policy. Similarly, in the PRP M/G/1 queuing network, when $\rho_{p}$ lies on the left side of decision point B (i.e., $\rho_{p}<0.44$), the secondary connection will adopt the changing policy. On the contrary, when $\rho_{p}$ lies on the right side of decision point B (i.e., $\rho_{p}>0.44$), the secondary connection will adaptively adopt the staying policy. In addition, the decision point A lies on the right side of the decision point B for the reason that the repeat scheduling discipline results in larger delay in the PRI M/G/1 queuing network than the resume scheduling discipline does in the PRP M/G/1 queuing network.

**Figure 7:**
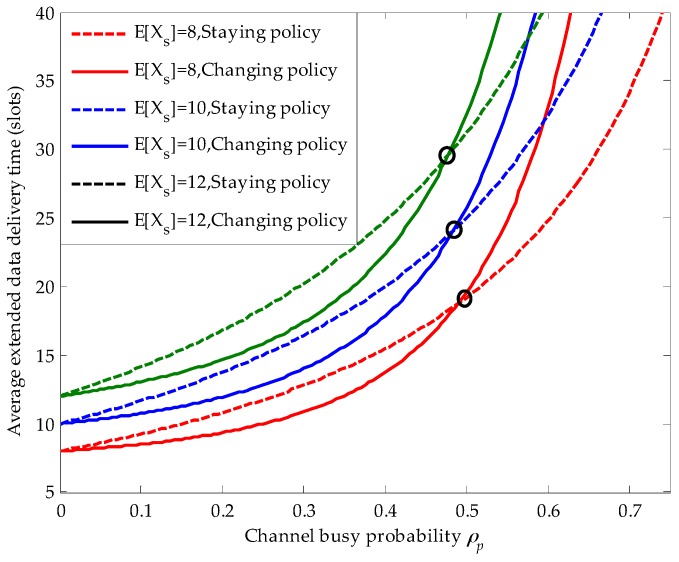
The effects of the secondary connection average service time on the average extended data delivery time in the preemptive repeat identical (PRI) M/G/1 queuing network, where the primary connection follows the exponential distribution, $\lambda_{s}=0.01$ (arrivals/slot), $\lambda_{p}=0.02$ (arrivals /slot), $0\leq \rho_{p}<0.75$, $t_{s}=1$ (slot), $E[X_{p}]=20$ (slots /arrival) and $\lambda_{s}E[X_{s}]+\rho_{p}<1$.

Figure 7 shows the effects of the secondary connection average service time on the average extended data delivery time and the position of the decision point in the PRI M/G/1 queuing network. Firstly, we can obviously see that with the increase of the secondary connection average service time, the average extended data delivery time becomes larger in both the staying and the changing policy. Because, the longer secondary connection average service time raises the risk that the ongoing secondary connection is interrupted by the newly arriving primary connections. Secondly, when the secondary connection average service time becomes larger, the corresponding decision point moves slightly towards left. For example, for $E[X_{s}]=8$ (slots), the horizontal coordinate of the corresponding decision point is 0.495, whereas for $E[X_{s}]=12$ (slots), the horizontal coordinate of the corresponding decision point moves to 0.475.

### 4.4. Admission Control Rule

By using the derived results in Section 3, we can design the admission control rule for the SU subject to its maximum cumulative delay requirement. The cumulative delay is referred to as the difference between the average extended data delivery time and the average service time of the secondary connection. Let *s* (slots) denote the maximum allowable delay for the SU. When the service time distribution of the primary connection follows the upper-truncated Pareto distribution, the optimal policy for the SU is to execute the staying policy based on above-mentioned analysis results. For a stable queuing system, we have:

(39) $$\left\{ \begin{array}{l} \frac{1}{1-\rho_{p}}\left(E\left[X_{s}\right]+\frac{1-p_{s}}{p_{s}}\frac{E\left[{\left(X_{s}\right)}^{2}\right]}{2E\left[X_{s}\right]}\right)-E\left[X_{s}\right]<s\\ 0\leq \rho_{s}+\rho_{p}<1\;\\ 0\leq \rho_{s}<1\\ 0\leq \rho_{p}<1 \end{array}\right.$$

**Figure 8:**
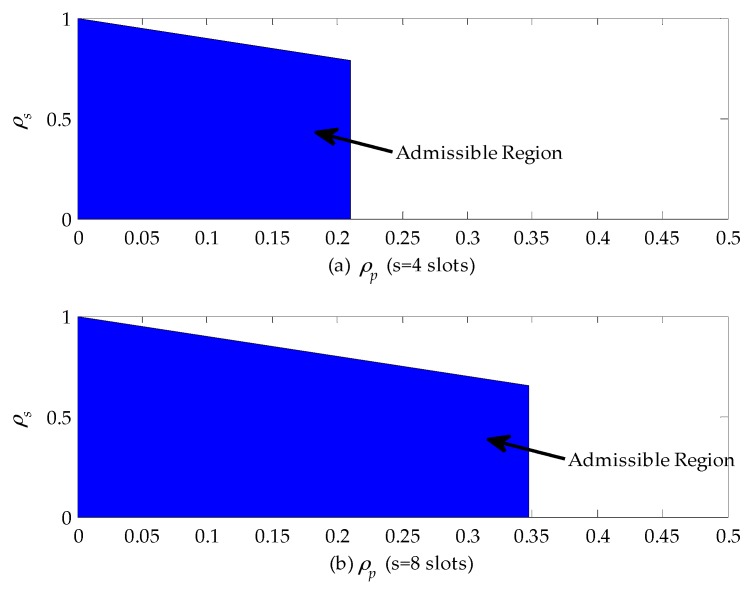
The admissible region of a cognitive radio (CR) network when the service time of the primary connection follows the upper-truncated Pareto distribution: (**a**) the maximum allowable delay is set to be four slots; and (**b**) the maximum allowable delay is set to be eight slots.

Figure 8 shows the admissible region of a CR network when the service time of the primary connection follows the upper-truncated Pareto distribution and the admission control policy can be designed based on these results. From Figure 8a, we can find that when $\rho_{p}\leq 0.2$, the CR can accept all secondary connection requests as long as they satisfy the queue stable condition, i.e., $0\leq \rho_{s}+\rho_{p}<1$. In the case that $\rho_{p}>0.2$, the CR network will reject any secondary connection request, because the delay is only related to channel busy probability $\rho_{p}$ for given $E[X_{s}]=10$ (slots) and $E[X_{p}]=20$ (slots) and exceeds the maximum allowable delay according to Equation (39). In addition, by comparing Figure 8a,b, we can obviously see that the horizontal coordinate of the admissible region extends from 0.2 to 0.345. That is because the maximum allowable delay is set to be eight slots, which is two times as large as before. Therefore, the secondary connection request can be accepted as long as the channel busy probability $\rho_{p}$ is no larger than 0.345.

When the service time distribution of the primary connection follows the exponential distribution, the optimal policy for the SU is to adopt the traffic-adaptive policy. Thus, for a stable queuing system, we have:

(40) $$\left\{ \begin{array}{l} \min\left(E\left[D\right],\frac{1}{1-\rho_{p}}\left(E\left[X_{s}\right]+\frac{1-p_{s}}{p_{s}}\frac{E\left[{\left(X_{s}\right)}^{2}\right]}{2E\left[X_{s}\right]}\right)-E\left[X_{s}\right]\right)<s\\ 0\leq \rho_{s}+\rho_{p}<1\;\\ 0\leq \rho_{s}<1\\ 0\leq \rho_{p}<1 \end{array}\right.$$

**Figure 9:**
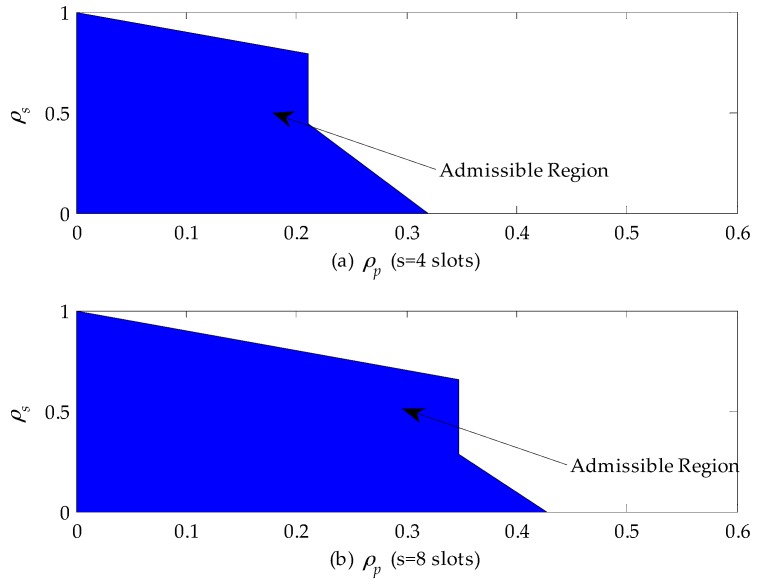
The admissible region of a cognitive radio (CR) network when the service time of the primary connection follows the exponential distribution: (**a**) the maximum allowable delay is set to be four slots; and (**b**) the maximum allowable delay is set to be eight slots.

Figure 9 shows the admissible region of a CR network when the service time of the primary connection follows the exponential distribution. From Figure 9a, we can find that if $\rho_{p}\leq 0.21$, the CR network can accept all secondary connection requests as long as they satisfy the queuing stable condition, i.e., $0\leq \rho_{s}+\rho_{p}<1$. If $0.21<\rho_{p}\leq 0.32$, the secondary connection requests can be accepted only when $\rho_{s}\leq 0.44$. Otherwise, they will be rejected to guarantee the maximum allowable delay of SUs. In addition, if $\rho_{p}>0.32$, no secondary connection can be built. That is because the delay of SUs exceeds the maximum allowable delay. Furthermore, comparing Figure 9a,b, we can find that a longer maximum allowable delay extends the admissible region. For instance, the horizontal coordinate of the admissible region extends from 0.32 to 0.43. When $\rho_{p}>0.43$, no SU is allowed to access to the CR network.

## 5. Conclusions

In this paper, an analytical framework model is proposed to describe spectrum handoff behaviors of the SU based on the PRI M/G/1 queuing network. Then, we introduce the staying and the changing scenarios and derive the close-expression of extended data delivery and sojourn time in both scenarios. Based on the derivation results, we propose the traffic-adaptive policy. That is, when the service time distribution of the primary connection follows the upper-truncated Pareto distribution, the best policy for the SU is to execute the staying policy. When the service time distribution of the primary connection follows the exponential distribution, the best policy for the SU is to choose a policy according to the channel probability $\rho_{p}$. In the case that $\rho_{p}<0.485$, the SU will choose to execute the changing policy. Otherwise, the staying policy will be the better policy for the SU. In addition, when the service time distribution of the secondary connection follows different distributions (i.e., the upper-truncated Pareto distribution and the exponential distribution), the simulation results are the same, because the average service times of these two distributions are set to be identical for fair comparison. Finally, we investigate the admissible region for a CR network and provide a reference for the arriving SU. Although the repeat scheduling discipline will result in larger delay in the PRI M/G/1 queuing network than the resume scheduling discipline does in the PRP M/G/1 queuing network, the repeat scheduling discipline is usually applied in some special scenarios. Thus, our work makes the research on spectrum handoff behaviors much more complete in CR network. Moreover, our work can provide the reference for executing the optimal spectrum handoff strategy and designing the admission control rule for the SU in the PRI M/G/1 queuing network.

Some important issues can also be considered in the future. For example, the energy efficiency problem in the PRI M/G/1 and the PRP M/G/1 queuing network is worth investigating. That is, the interrupted SU should execute the optimal spectrum handoff strategy to maximize the energy efficiency during the whole data transmission process.
